# Antiseptic Surgery

**Published:** 1881-11-20

**Authors:** 


*Antiseptic Surgery.*—The principles, modes of application and results of the Lister Dressing, by Dr. Just Lucas, Championiere Surgeon to the Hospital Tenon, Member of the Societe de Chirurgie, Editor of the Journal de Medicine et de Chirurgie pretiques. Translated from the second and completely revised edition, with the special sanction of the author, and edited by Frederick Henry Gerrish, A. M. M. D., Surgeon to the Maine General Hospital, Prof. of Mat. Medica and Therapeutics in Bowden College, etc.

Portland—Loring, Short & Hammond; a work of 239 oc. pages, very recent, full and complete on the subject treated.

